# Human pluripotent stem cell-derived cells endogenously expressing follicle-stimulating hormone receptors: modeling the function of an inactivating receptor mutation

**DOI:** 10.1093/molehr/gaac012

**Published:** 2022-04-26

**Authors:** K Lundin, K Sepponen, P Väyrynen, X Liu, D A Yohannes, M Survila, B Ghimire, J Känsäkoski, S Katayama, J Partanen, S Vuoristo, P Paloviita, N Rahman, T Raivio, K Luiro, I Huhtaniemi, M Varjosalo, T Tuuri, J S Tapanainen

**Affiliations:** 1 Department of Obstetrics and Gynecology, University of Helsinki and Helsinki University Hospital, Helsinki, Finland; 2 Molecular Systems Biology Research Group, Institute of Biotechnology & HiLIFE, University of Helsinki, Helsinki, Finland; 3 Proteomics Unit, Institute of Biotechnology & HiLIFE, University of Helsinki, Helsinki, Finland; 4 Research Programs Unit, Translational Immunology & Department of Medical and Clinical Genetics, University of Helsinki, Helsinki, Finland; 5 Molecular and Integrative Biosciences Research Programme, Faculty of Biological and Environmental Sciences, University of Helsinki, Helsinki, Finland; 6 Institute for Molecular Medicine Finland, University of Helsinki, Helsinki, Finland; 7 Department of Physiology, University of Helsinki, Helsinki, Finland; 8 Folkhälsan Research Center, Helsinki, Finland; 9 Department of Biosciences and Nutrition, Karolinska Institutet, Huddinge, Sweden; 10 Stem Cells and Metabolism Research Program, Faculty of Medicine, University of Helsinki, Helsinki, Finland; 11 Institute of Biomedicine, University of Turku, Turku, Finland; 12 Department of Reproduction and Gynecological Endocrinology, Medical University of Bialystok, Bialystok, Poland; 13 New Children's Hospital, Pediatric Research Center, Helsinki University Hospital, HUH, Helsinki, Finland; 14 Department of Metabolism, Endocrinology and Reproduction, Faculty of Medicine, Hammersmith Campus, Imperial College London, London, UK; 15 Department of Obstetrics and Gynecology, University Hospital of Oulu, University of Oulu, Medical Research Center Oulu and PEDEGO Research Unit, Oulu, Finland

**Keywords:** FSH action, G-protein coupled receptor function, receptor mutation, infertility, pluripotent stem cell

## Abstract

Follicle-stimulating hormone (FSH) is crucial in the development and regulation of reproductive functions. The actions of human FSH and its receptor (FSHR) and mutations therein have mainly been studied using *in vivo* models, primary cells, cancer cells and cell lines ectopically expressing the FSHR. To allow studies of endogenous FSHR function *in vitro*, we differentiated FSHR-expressing cells from human pluripotent stem cells. FSH stimulation of the wild-type (WT), but not the inactivating Finnish founder mutant (A189V) receptor, activated the canonical cyclic adenosine monophosphate (cAMP)-dependent signaling pathway and downstream mediators. To investigate protein–protein interaction partners of FSHR at resting state and upon FSH stimulation, we expressed FSHR in HEK293 cells followed by affinity purification mass spectrometry analyses. We found 19 specific high-confidence interacting proteins for WT FSHR and 14 for A189V FSHR, several of which have been linked to infertility. Interestingly, while only WT FSHR interacted with FSH, insulin-like growth factor 1 receptor (IGF1R), for example, interacted with both WT and A189V FSHR upon FSH stimulation. In conclusion, our protocol allows detailed studies of FSH action and disease modeling in human cells endogenously expressing FSHR.

## Introduction

Follicle-stimulating hormone (FSH) is a heterodimeric glycoprotein hormone secreted by the gonadotroph cells of the anterior pituitary gland. It consists of a common α-subunit (CGA) shared with other glycoprotein hormones, and a specific β-subunit (FSHB) that confers specificity for the FSH receptor (FSHR) (Papkoff and Ekblad, 1970; Boime and Ben-Menahem, 1999). FSHR belongs to the class A G-protein coupled receptor (GPCR) superfamily, expressed predominantly on the plasma membrane of Sertoli cells of the testes and granulosa cells of the ovaries (Simoni *et al.*, 1997). In addition, low FSHR expression has lately been reported in a variety of extragonadal tissues (Kumar, 2018; Chrusciel *et al.*, 2019; Lizneva *et al.*, 2019). In males, FSH is essential for normal testicular function and it supports growth and maturation of the Sertoli cells, and thereby indirectly spermatogenesis (Plant and Marshall, 2001; Oduwole *et al.*, 2018). In females, FSH is mandatory for ovarian follicular growth and maturation, as well as estrogen production (Aittomaki *et al.*, 1995; Kumar *et al.*, 1997). Various inhibiting mutations in *FSHR* have been shown to block normal oocyte maturation, resulting in primary ovarian insufficiency due to arrested follicular growth at the antral stage (Aittomaki *et al.*, 1995; Desai *et al.*, 2013; Liu *et al.*, 2017). A recent clinical study showed a similar cardiovascular and metabolic risk profile in women with an inactivating *FSHR* mutation compared to population controls, but suboptimal bone health despite estrogen treatment (Luiro *et al.*, 2019). In men, inactivating mutations in *FSHR* lead to subfertility due to oligozoospermia, but not to complete infertility (Tapanainen *et al.*, 1997; Simoni *et al.*, 2016). In both men and women, however, mutations in FSHB lead to infertility (Matthews *et al.*, 1993; Layman *et al.*, 1997; Huhtaniemi, 2006; Zheng *et al.*, 2017). The reason for the discrepant phenotypes between FSH ligand and receptor inactivation remains unclear. Conspicuously, both *Fshr* and *Fshb* knockout male mice are fertile with only a slight reduction of spermatogenesis (Kumar *et al.*, 1997; Dierich *et al.*, 1998; Abel *et al.*, 2000).

Binding of FSH to the FSHR activates different signaling pathways, resulting in gene transcription, steroidogenesis, proliferative and pro- as well as anti-apoptotic signals, depending on cell type and stage (Sayers and Hanyaloglu, 2018; Casarini and Crepieux, 2019). In the classical FSHR-mediated pathway, ligand binding results in activation of adenylyl cyclase by the stimulatory Gαs subunit, subsequently leading to cyclic AMP (cAMP) production and activation of the protein kinase A signaling pathway and its downstream mediators extracellular-regulated kinase 1 and 2 (ERK1/2), cAMP-responsive elements binding protein and the p38 MAPK pathway (Means *et al.*, 1974; Azhar *et al.*, 1976; Ulloa-Aguirre *et al.*, 2007). Ligand binding also activates other G proteins, such as the inhibitory Gαi subunit activating ERK1/2 signaling (Davenport and Heindel, 1987; Crepieux *et al.*, 2001) and Gαq that activates phospholipase C-mediated protein kinase C, IP_3_ and Ca^2+^-mediated signaling pathways (Lin *et al.*, 2006; Escamilla-Hernandez *et al.*, 2008; Sanchez-Fernandez *et al.*, 2014). β-arrestins, typically mediating ligand-induced FSHR desensitization and recycling, also activate ERK1/2 signaling in a G-protein independent, delayed and sustained fashion (Crepieux *et al.*, 2001; Kara *et al.*, 2006; Sayers and Hanyaloglu, 2018), especially in cells with low receptor density (Tranchant *et al.*, 2011; Casarini *et al.*, 2016).

The function and regulation of FSH and FSHR interaction have been studied mainly using *invivo* models and primary cells, as well as tumor and immortalized cell lines overexpressing *FSHR* (Rannikko *et al.*, 2002; Tranchant *et al.*, 2011; Casarini *et al.*, 2016). In primary cells, endogenous *FSHR* is rapidly downregulated *in vitro* and, upon transfection, the high *FSHR* expression is driven by unphysiological (usually viral) promoters which may compromise the functional meaning of the results (Rannikko *et al.*, 2002; Tranchant *et al.*, 2011; Casarini *et al.*, 2016). The function of the receptor gene promoter cannot be addressed in such cell models. In addition to FSH concentration, the density of FSHR has been shown to be a quintessential determinant of the type of signaling cascades activated (Dong *et al.*, 2007; Tranchant *et al.*, 2011; Sayers and Hanyaloglu, 2018). Furthermore, it has been suggested that FSHR variants are expressed differently in diverse cell types and even during the developmental stages of gonadal cells, providing potential explanations for their differential responses to FSH stimulation (Kumar, 2018; Chrusciel *et al.*, 2019; Lizneva *et al.*, 2019). This emphasizes the biological validity of the cell models in the study of receptor function.

The aim of this study was to generate a protocol to differentiate human embryonic stem cells (hESCs) and patient-derived human-induced pluripotent stem cells (hiPSCs) into cells endogenously expressing *FSHR*, to enable studies of the receptor function in more physiological conditions. In addition, we have specifically addressed the function of the inactivating A189V mutation in *FSHR*, a mutation that presumably results in sequestering of the receptor inside the cell (Aittomaki *et al.*, 1995; Tapanainen *et al.*, 1997; Rannikko *et al.*, 2002). Given the potential of human pluripotent stem cells (hPSCs) to differentiate into any cell type of the human body, this system also opens up the possibility to study the role of FSH/FSHR in different biological contexts.

## Materials and methods

### Ethical statement

This study as well as generation of the hiPSC lines used in the study was approved by the Coordinating Ethics Committee of the Helsinki and Uusimaa Hospital District (HUS/2064/2019, 333/13/03/03/2013) with informed consent from the donors.

### Maintenance and differentiation of human pluripotent stem cells

Human ESC line H9 (WA09, 46,XX; WiCell Research Institute, Madison, WI, USA) (Thomson *et al.*, 1998) was used as a wild-type (WT) control line. Cells were maintained on hESC-qualified Geltrex in Essential 8™ medium, split every 4–5 days in a 1:4–1:10 ratio using 0.5 mM EDTA in phosphate-buffered saline solution (PBS; all from Thermo Fisher Scientific, Waltham, MA, USA). All cells were cultured in a humidified incubator supplied with 5% CO_2_ at 37°C and regularly checked for Mycoplasma contamination.

For differentiation, cells at 80% confluency were washed once with PBS and dissociated into single cells using StemPro™ Accutase™ Cell Dissociation Reagent (Thermo Fisher Scientific) for 5 min at 37°C. Cells were resuspended in differentiation medium containing DMEM/F12 + Glutamax supplemented with 2% B27™ Supplement (Thermo Fisher Scientific). Cells were counted, centrifuged for 5 min at 70×*g* and resuspended in differentiation medium containing 10 µM Rho kinase inhibitor (Y-27632 2HCl, Selleckchem, Houston, TX, USA), 100 ng/ml Activin A (Q-kine, Cambridge, UK), and 5 µM GSK-3α/β inhibitor CHIR-99021 (Selleckchem). Then, 1.5 × 10^5^ cells/cm^2^ were plated in 12-well plates coated with 0.5 µg/cm^2^ human Collagen I (Corning, NY, USA). The following day, the medium was changed into differentiation medium containing 3 µM CHIR-99021 and 50 ng/ml bone morphogenetic protein (BMP) 7 (Peprotech, Cranbury, NJ, USA) and incubated for 24 h, whereafter differentiation medium containing 3 µM CHIR and 2 µM Dorsomorphin (Selleckchem), a small molecule BMP inhibitor, was kept for 48 h. The cells were further differentiated in differentiation medium without supplements until Day 8.

### Generation of human-induced pluripotent stem cell lines carrying A189V mutation in FSHR

Fibroblasts were obtained from skin biopsy of two women with FSH-resistant ovaries (FSHRO) caused by an inactivating A189V mutation in the *FSHR* gene. The women were aged 32 (cell line HEL127.6) and 56 (cell line HEL128.5) years and were undergoing no hormonal treatment. The cells were reprogrammed using episomal vectors containing reprogramming factors OCT3/4, LIN28, KLF4, SOX2, L-MYC and shRNA against p53 and characterized to meet the common criteria of iPSCs at Biomedicum Stem Cell Center (BSCC, Helsinki, Finland) as a local core service. The two patient-derived A189V mutant cell lines, HEL127.6 and HEL128.5, used in this study, were maintained and differentiated as described above for hPSCs.

### Single-cell sequencing and analysis

#### Cell dissociation

WT cells at Day 8 of differentiation from two separate experiments were used for Chromium Single Cell Gene Expression analysis. The cell dissociation procedure was modified from previously published protocols (Klein *et al.*, 2015; Balboa *et al.*, 2018). Cells were first washed twice with Dulbecco’s PBS (without Mg^2+^/Ca^2+^) and dissociated into single cells with 1:1 mixture of Trypsin-EDTA and TrypLE™ Express (all from Thermo Fisher Scientific) for 10 min at 37°C. Dissociated cells were resuspended into DMEM/F12 + 2% B27 and mixed well, before passing through a 40 µm cell strainer (Falcon, Thermo Fisher Scientific) to remove cell clumps. Excess floating RNA was removed by harvesting cells at 300×*g* for 5 min and resuspending them into Dulbecco’s PBS (without Mg^2+^/Ca^2+^) containing 0.04% bovine serum albumin (BSA; Sigma-Aldrich, St. Louis, MO, USA). Cells were gently mixed by pipetting five times and harvesting was repeated at 300×*g* whereafter the pellet was resuspended in cold 0.04% BSA in PBS by carefully pipetting 15 times. Cells were passed through a cell strainer once more to minimize the number of cell doublets. Cells were kept on ice until analysis and cell viability% and doublet% were evaluated using LUNA-STEM Automated Fluorescence Cell Counter (Logos Biosystems; South Korea). Cell viability was above 90% and the doublets were below 8% in both samples.

#### Single-cell mRNA library preparation and sequencing

Library preparation, sequencing as well as preliminary data processing were performed at the Institute for Molecular Medicine Finland (FIMM, Helsinki, Finland). Single-cell gene expression profiles were studied using 10× Genomics Chromium Single Cell 3′RNASeq platform. The Chromium Single Cell 3′RNAseq run and library preparations were done using the Chromium Next GEM Single Cell 3′Gene Expression version 3.1 Dual Index chemistry. The Sample libraries were sequenced on Illumina NovaSeq 6000 Sequencing system (Illumina, San Diego, CA, USA) using read lengths: 28 bp (Read 1), 10 bp (i7 Index), 10 bp (i5 Index) and 90 bp (Read 2).

#### Data analysis

Preliminary data processing and analysis were performed using 10× Genomics Cell Ranger (version 6.0.0) count and aggr pipelines. Combined filtered data from Cell Ranger aggr pipeline contained in total 12 201 cells and 36 601 genes.

Downstream data analysis was done using Seurat (version 4.0.3; http://seurat.r-forge.r-project.org) (Butler *et al.*, 2018), along with R (version 4.1.0; https://www.r-project.org/). Cells having <3 genes and genes detected in <200 cells were discarded from the analysis. Furthermore, cell having more than 10% mitochondrial gene count and overall gene counts <15 or >9000 were filtered out. After filtering, 23 883 genes and 11 906 cells remained for further analysis. Before performing principal component analysis (PCA), data were normalized using sctransform and effect coming from mitochondrial genes was regressed out. First 35 principal components were used in clustering and UMAP (Uniform Manifold Approximation and Projection) visualization. R package scCATCH (version 2.1; https://github.com/ZJUFanLab/scCATCH) (Shao *et al.*, 2020) was used to annotate clusters to cell types using reference tissues related to kidney, gonad, embryo and fetus.

Details on read alignment can be found in Supplementary Table SI.

### FSH stimulation and cAMP detection

At Day 8 of differentiation, cells were washed with PBS (with Mg^2+^/Ca^2+^; Sigma-Aldrich) and incubated for 1 or 8 h at 37°C with 1, 10 or 100 ng/ml human recombinant FSH (Prospec, Rehovot, Israel), vehicle (0.1% BSA in H_2_O) or 10 µM forskolin (Sigma-Aldrich) in differentiation medium. For cAMP measurement, cells were stimulated for 1 h, lysed in HCl inactivating phosphodiesterases, and analyzed with Direct cAMP ELISA Kit (EnzoLife Sciences (ELS) AG, Lausen, Switzerland, Cat# ADI-900-066) following the manufacturer’s instructions. Phosphodiesterase inhibitors were not added during the stimulation. After blanking, the optical density was read at 405 nm using a Multiscan EX Version 1.1 (type 355; Labsystems, Vantaa, Finland) microplate reader and the standard curve was prepared using Point-to-Point method. For analyzing mRNA expression levels with qRT-PCR, cells were stimulated for 8 h and lysed in RA1 buffer (Macherey-Nagel, Duren, Germany) according to manufacturer’s instructions and frozen for later RNA extraction.

### RNA extraction and quantitative real-time RT-PCR

Total RNA was extracted from lysates collected at indicated days of differentiation and after FSH stimulation using NucleoSpin RNA kit (Macherey-Nagel). Residual genomic DNA was removed in a separate step using RQ1 DNase (Promega, Madison, WI, USA) and RiboLock RNase inhibitor (Fisher Scientific, Hampton, NH, USA) for 30 min at 37°C to ensure complete removal of genomic DNA, after which the samples were purified using RNA Cleanup kit (Macherey-Nagel). Samples were reverse transcribed into cDNA with Moloney murine leukemia virus reverse transcriptase (Promega), oligo(dT)18 primers, random hexamer primers, a mixture of four deoxynucleotide primers, and RiboLock RNase inhibitor (all from Fisher Scientific) for 90 min at 37°C. cDNA, forward and reverse primers (Metabion, Planegg/Steinkirchen, Germany) and HOT FIREPol EvaGreen qPCR Mix Plus (Solis Biodyne, Tartu, Estonia) were mixed and relative mRNA expression levels analyzed with LightCycler96 System (Roche Diagnostics, Mannheim, Germany). For relative quantification of gene expression, we followed the ΔΔCt method (Livak and Schmittgen, 2001). Expression levels were normalized using cyclophilin G (*PPIG*) as an endogenous control (Balboa *et al.*, 2015) and presented as relative to expression levels in undifferentiated cells. Primer sequences used: *FSHR* FWD 5′-ATC TGT CAC TGC TCT AAC AG GGT-3′ and REV 5′-TCT CCA GGT CCC CAA ATC CT-3′; *INHA* (Casagrandi *et al.*, 2003) FWD 5′-CTC GGA TGG AGG TTA CTC TTT CAA-3′ and REV 5′-GAA GAC CCC CCA CCC TTA GA-3′; *StAR* 5′-GAG TCA GCA GGA CAA TGG GG-3′ and REV 5′-CGC TCC ACG AGC TCT TCA TA-3′; *DLK1* FWD 5′-GGA CGG GGA GCT CTG TGA TA-3′ and REV 5′-CGT CCT TTT TCT GGC AGT CC-3′; *PPIG* (Balboa *et al.*, 2015) FWD 5′-TCT TGT CAA TGG CCA ACA GAG-3′ and REV 5′-GCC CAT CTA AAT GAG GAG TTG-3′.

### MAC-Tag protein–protein interaction

#### Generation of stable cell line

The PCR-product of A189V mutant FSHR obtained from HEL127.6 cells differentiated until Day 8 as described above was used to generate the Gateway compatible entry clone. To generate the Gateway entry clone, flanking attB sites were added to each end of the full-length *FSHR* A189V mutant cDNA by using PCR, followed by a Gateway BP reaction. The WT FSHR was prepared by correcting the A189V substitution back to the WT sequence by using the QuikChange II XL site-directed mutagenesis kit (Agilent Technologies, Santa Clara, CA, USA), in order to avoid effects of possible single-nucleotide polymorphisms. The integrity of the clones was verified by sequencing. Sequencing of the PCR product confirmed that the cells generated by our protocol expressed the full-length variant of the transcript. LR recombination was performed between the entry clones and the MAC-C destination vector (Liu *et al.*, 2018) to generate C-terminally MAC-tagged WT and A189V mutant FSHR expression vectors as described previously (Liu *et al.*, 2020). MAC-tagged green fluorescent protein (GFP) expression vector was used as a control.

Flp-In™ 293 T-REx cells (Thermo Fisher Scientific, Cat# R78007) were co-transfected with the expression vector and the pOG44 vector (Thermo Fisher Scientific) using the DreamFect™ reagent (Oz Biosciences, San Diego, CA, USA) according to manufacturer’s instructions. Tetracycline-inducible Flp-In™ 293 T-REx cell lines expressing WT FSHR, A189V FSHR or GFP were generated as described in Varjosalo *et al.*, 2013. To study interactions with ligand-bound receptors, WT FSHR, A189V FSHR and GFP expressing cells were additionally incubated for 2 h at 37°C with 100 ng/ml human recombinant FSH (Prospec). A 2 h incubation was estimated to be enough to allow protein interactions to occur. Cells deriving from 5 × 150 mm fully confluent dishes (approximately 5 × 10^7^ cells) were pelleted as one biological sample. Each processed sample consists of three biological samples. Samples were frozen at −80°C until further use.

#### Purification and mass spectrometry

For affinity purification, pellets (∼5 × 10^7^ cells) of cells stably expressing MAC-tagged gene of interest or GFP control (three biological replicates) were lysed in 3 ml of lysis buffer (50 mM HEPES pH 8.0, 5 mM EDTA, 150 mM NaCl, 50 mM NaF, 0.5% NP40, 1 mM DTT, 1.5 mM Na_3_VO_4_, 1 mM PMSF, and 1× protease inhibitor cocktail; Sigma) on ice. Clear cell lysates were obtained by centrifugation and loaded to columns (Bio-Rad Laboratories, Hercules, CA, USA) containing 200 μl Strep-Tactin matrix (IBA, GmbH, Göttingen, Germany). Bound proteins were washed and finally eluted with 900 μl of elution buffer (0.5 mM Biotin; Pierce). The detailed procedures were as described (Liu *et al.*, 2020). Purified protein complexes were reduced with 5 mM TCEP (Tris (2-carboxyethyl) phosphine) and alkylated for cysteine bonds with 10 mM iodoacetamide. Proteins were digested with trypsin (Promega) overnight at 37°C. The tryptic peptides were desalted using C-18 microspin columns (The Nest Group Inc., Southboro, MA, USA) as per manufacturer’s instructions, and processed for mass spectrometry analysis. The analysis was performed on a Q-Exactive mass spectrometer using Xcalibur version 3.0.63 coupled with an EASY-nLC 1000 system via an electrospray ionization sprayer (Thermo Fisher Scientific) (Liu *et al.*, 2020).

#### Data processing

For protein identification, Thermo .RAW files were uploaded into Proteome Discoverer 1.4 (Thermo Fisher Scientific) and searched with Sequest search engine against the selected human component of UniProtKB/SwissProt database (http://www.uniprot.org). All reported data were based on peptides assigned in Proteome Discoverer (Thermo Fisher Scientific) with a 5% false discovery rate (FDR) by Percolator. The high-confidence (protein–protein) interactions (HCIs) were identified using stringent filtering against GFP control samples and the Contaminant Repository for Affinity Purification (CRAPome, http://www.crapome.org/) (Mellacheruvu *et al.*, 2013) database. HCIs data were imported into Cytoscape 3.4.0 (http://cytoscape.org) for visualization. Gene ontology classification analysis was based on the DAVID bioinformatics resource (https://david.ncifcrf.gov/) (Huang *et al.*, 2009) and literature mining. A search on male and female infertility gene sets was performed for all HCIs in the curated Comparative Toxicogenomics Database (CTD) Gene-Disease Associations (Davis *et al.*, 2009) dataset using the Harmonizome (http://amp.pharm.mssm.edu/Harmonizome/) (Rouillard *et al.*, 2016).

#### Statistical analyses

Significance Analysis of INTeractome (SAINT) (Choi *et al.*, 2011) and Contaminant Repository for Affinity Purification (Mellacheruvu *et al.*, 2013) (CRAPome, http://www.crapome.org/) were used as statistical tools for probabilistically scoring protein–protein interaction (PPI) data and identifying specific high-confidence interactions from our affinity purification-mass spectrometry (AP-MS) data. A set of 26 GFP control runs (13 N-terminal MAC-GFP and 13 C-terminal MAC-GFP) were used as control counts for each hit and the final results only considering proteins with Bayesian FDR < 0.01. Furthermore, any proteins that were detected in CRAPome database with a frequency of ≥20% were eliminated.

Statistical analyses for cAMP and qRT-PCR data were conducted with IBM^®^ SPSS^®^ Statistics 28 software. cAMP data were analyzed with two-way analysis of variance (ANOVA) followed by Bonferroni multiple comparison adjustment. The gene expression data were analyzed with one-way ANOVA or independent samples Kruskal–Wallis test with Bonferroni correction for multiple comparisons, or with independent samples *t*-test (two-sided) according to the sample set. Benjamini and Hochberg method was used to adjust *P*-values of multiple independent samples *t*-tests. Shapiro–Wilk test was used to assess normal distribution and Levene’s test the homogeneity of variances. When assumptions for ANOVA were not met, Kruskal–Wallis test was used for adjusting multiple comparisons. *P *<* *0.05 was considered to indicate statistical significance. All statistical analyses were performed at confidence level 95%.

## Results

### Generation of cells expressing functional FSHR

**Figure 1.**
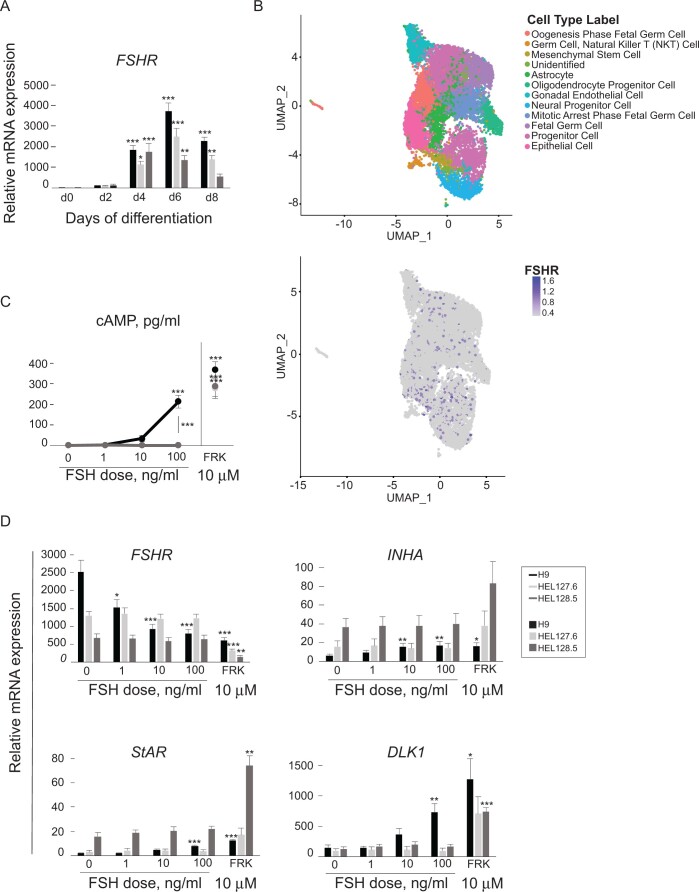
**Characterization of differentiated H9 (wild-type) human embryonic stem cells (hESCs) and HEL127.6 and HEL128.5 (A189V FSHR mutant) patient-derived human embryonic stem cells (hiPSCs).** (**A**) FSHR expression was detected from Day (d) 2 of differentiation by quantitative RT-PCR when compared with that in undifferentiated cells (d0). (**B**) Single-cell RNA sequencing followed by cluster-based automatic cell annotation predicted 11 clusters with identified cell types and 1 unidentified cluster. FSHR expressing cells (blue dots) were detected in all annotated clusters. (**C**) Wild-type (WT) cells (black line) at Day 8 of differentiation responded to FSH stimulation by cyclic AMP (cAMP) production. A189V FSHR mutant cells at Day 8 of differentiation (light and dark gray lines) did not respond to FSH stimulation by cAMP production, but all cell lines produced cAMP upon forskolin treatment. *X*-axis indicates FSH concentrations or forskolin used for stimulation. 0 ng/ml = vehicle control, FRK = 10 µM forskolin. (**D**) FSH stimulation for 8 h on Day 8 of differentiation reduced *FSHR* expression and increased expression of Inhibin α subunit (*INHA*), Steroidogenic Acute Regulatory Protein (*StAR*) and Delta Like Non-Canonical Notch Ligand (*DLK1*) in WT cells (black bars) but not in A189V mutant lines (light and dark gray bars). All cells responded to stimulation with 10 µM forskolin. The values represent mean ± SEM from six (WT), seven (HEL127.6) or four (HEL128.5) (A), four (WT and A189V) (C) and three or six (WT and A189V) (D) independent experiments, respectively. Black bars and line; H9 WT cells; light gray bars and line: HEL127.6 A189V mutant cells; dark gray bars and line: HEL128.5 A189V mutant cells. *P*-values were calculated in (A) with one-way ANOVA followed by Bonferroni test for multiple comparison adjustment, in (C) with two-way ANOVA followed by Bonferroni test for multiple comparison adjustment, in (D) *FSHR*, *StAR* and *DLK1* FSH stimulations with one-way ANOVA followed by Bonferroni test for multiple comparison adjustment, in *INHA* FSH stimulations with independent samples Kruskal–Wallis test with Bonferroni correction for multiple comparison adjustment, and in all forskolin stimulations with independent samples *t*-test (two-sided). *P <* *0.05, **P <* *0.01, ***P <* *0.001.

To generate cells endogenously expressing FSHR, H9 cells (control WT hESC line) were differentiated utilizing a protocol slightly modified from our previously published protocols for differentiation of bipotential gonad-like cells (Sepponen *et al.*, 2017). Here, the concentration of BMP7 was increased and BMP signaling was inhibited only after the primitive streak stage, in order to enrich FSHR expression. The *FSHR* mRNA expression was detected already at Day 2 of differentiation and it typically peaked at Day 6, whereafter the mRNA levels decreased when compared with undifferentiated cells [ANOVA, Bonferroni test, *P* (H9 Day 4) = 0.0003, *P* (H9 Day 6) = 5.31E−11, *P* (H9 Day 8) = 4E−06, n = 6 experiments] (Fig. 1A). To identify the differentiated cells, we performed single-cell RNA sequencing (scRNAseq) analysis. The annotation of several of the cluster was related to gonadal cell types (oogenesis phase fetal germ cells, gonadal endothelial cells, mitotic arrest phase fetal germ cells, fetal germ cells) and many of the clusters also associated with fetal, stem cell or progenitor stages of cells, when using single-cell Cluster-based Automatic Annotation Toolkit for Cellular Heterogeneity (csCATCH) for cell type prediction (Fig. 1B). Overall, the cells were very homogeneous forming overlapping clusters with FSHR expressing cells present in all annotated clusters, the overall percentage of FSHR expressing cells being 7.9 (Fig. 1B).

### Functionality of cells expressing wild-type FSHR

The functionality of the FSHR was tested at Day 8 of differentiation by measuring cAMP response after 1 h stimulation with 1, 10 or 100 ng/ml of recombinant human FSH, or with 10 µM forskolin that directly activates adenylyl cyclase. A clear dose-dependent increase in cAMP was detected when compared with vehicle as shown in Fig. 1C [ANOVA, Bonferroni test, *P* (FSH 10 ng/ml) = 0.116, *P* (FSH 100 ng/ml) = 1.58E−23, *P* (forskolin) = 1.75E−09, n = 4 experiments]. In addition, we measured the mRNA expression levels of a few selected target genes known to be regulated by FSH. After 8 h of FSH stimulation, the cells responded by dose-dependent downregulation of *FSHR* mRNA expression [FSH stimulations ANOVA, Bonferroni test, *P* (FSH 1 ng/ml) = 0.014, *P* (FSH 10 ng/ml) = 3.3E−05, *P* (FSH 100 ng/ml) = 9E−06, forskolin *t*-test, adjusted *P *=* *0.0005, n = 6 experiments] as previously demonstrated for *invitro* conditions, whereas the level of inhibin α subunit (*INHA*) mRNA was slightly upregulated [FSH stimulations Kruskal–Wallis test, adjusted *P* (FSH 10 ng/ml) = 0.003, *P* (FSH 100 ng/ml) = 0.002, forskolin *t*-test, adjusted *P *=* *0.03, n = 6 experiments] (Fig. 1D). There was no change in the expression level of inhibin β subunit in any of the conditions including forskolin stimulation (Supplementary Fig. S1). The expression of steroidogenic acute regulatory protein (*StAR*) and delta like non-canonical notch ligand 1 (*DLK1*) were also stimulated by FSH and forskolin (Fig. 1D) [FSH stimulations ANOVA, Bonferroni test, *P* (StAR, FSH 10 ng/ml) = 0.068, *P* (StAR, FSH 100 ng/ml) = 4.6E−05, *P* (DLK1, FSH 10 ng/ml) = 0.754, *P* (DLK1, FSH 100 ng/ml) = 0.002, forskolin *t*-test, adjusted *P* (StAR) = 1.34E−08, *P* (DLK1) = 0.038, n = 3 experiments].

### Functionality of cells expressing A189V mutant FSHR

To study cells expressing the mutant A189V FSHR, two hiPS cell lines, HEL127.6 and HEL128.5, were derived from skin fibroblasts of two female patients diagnosed with FSHRO, a syndrome carrying homozygous A189V mutation in the *FSHR* gene (Aittomaki *et al.*, 1995; Luiro *et al.*, 2019). There was no morphological difference between the WT and mutant cells upon differentiation, and also the cells carrying the A189V mutation expressed *FSHR* when compared with undifferentiated cells of the same line, albeit at a slightly lower level than the control cell line (Fig. 1A) [ANOVA, Bonferroni test, *P* (HEL127.6 Day 4) = 0.032, *P* (HEL127.6 Day 6) = 1.14E−07, *P* (HEL127.6 Day 8) = 0.0016, *P* (HEL128.5 Day 4) = 0.0003, *P* (HEL128.5 Day 6) = 0.0016, *P* (HEL128.5 Day 8) = 0.656, n (HEL127.6) = 7 experiments, n (HEL128.5) = 4 experiments]. Expression levels of genes typically vary between hPSC lines. The functionality of the mutant FSHR was tested on Day 8 of differentiation by stimulating the cells for 1 or 8 h with increasing doses of FSH or forskolin. In contrast to the WT cells, the cells carrying the mutation failed to respond to FSH stimulation by cAMP production at any dose, whereas forskolin induced cAMP production when compared to vehicle (Fig. 1C) [ANOVA, Bonferroni test, *P* (HEL127.6 forskolin) = 9.37E−08, *P* (HEL128.5 forskolin) = 8.61E−08, *P* (H9vsHEL127.6 10 ng/ml FSH) = 0.057, *P* (H9vsHEL127.6 100 ng/ml FSH) = 7.35E−24, *P* (H9vsHEL128.5 10 ng/ml FSH) = 0.0695, *P* (H9vsHEL128.5 100 ng/ml FSH) = 9.2E−24, n = 4 experiments]. The A189V mutant cells also failed to respond to eight hours of FSH stimulation at transcriptional level, with no change in *FSHR*, *INHA*, *StAR* nor *DLK1* mRNA levels. However, the cells responded to forskolin also at transcriptional level (Fig. 1D) [forskolin *t*-test, adjusted *P* (HEL127.6 FSHR) = 4.5E−05, *P* (HEL127.6 INHA) = 0.265, *P* (HEL127.6 StAR) = 0.11, *P* (HEL127.6 DLK1) = 0.117, *P* (HEL128.5 FSHR) = 0.0075, *P* (HEL128.5 INHA) = 0.172, *P* (HEL128.5 StAR) = 0.0011, *P* (HEL128.5 DLK1) = 0.0005, n (HEL127.6 FSHR) = 6 experiments, n (HEL127.6 StAR, DLK1) = 3 experiments, n (HEL127.6 INHA) = 4 experiments, n (HEL128.5) = 3 experiments].

### A189V mutation in FSHR affects protein interaction

**Figure 2.**
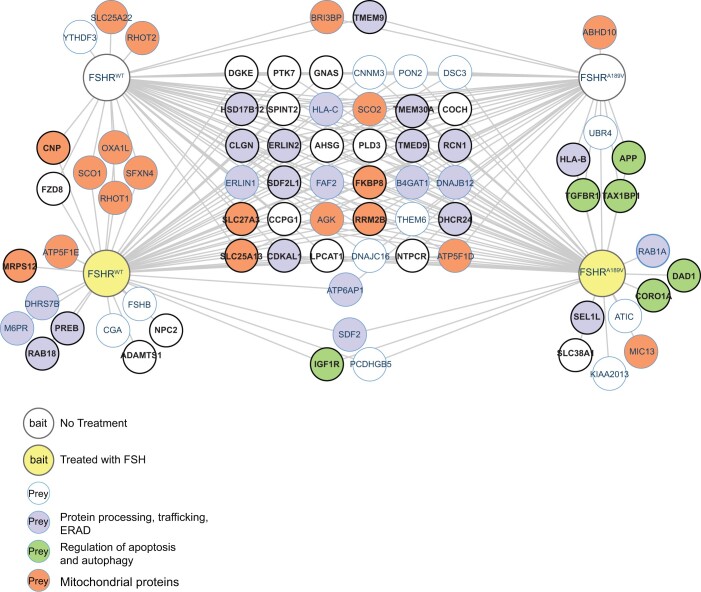
**Protein–protein interaction map of wild-type and A189V FSH receptor (FSHR) with or without FSH stimulation.** Affinity purification mass spectrometry (AP-MS) analysis of proteins interacting with non-stimulated (upper, white bait nodes) or FSH-stimulated (lower, yellow bait nodes) wild-type (WT) or A189V FSHR expressed in HEK293 cells. Prey nodes indicate proteins interacting with each of the four baits, colored according to localization or function. FSHR^WT^ = wild type FSHR; FSHR^A189V^ = A189V mutant FSHR. Bolded black protein names and node edges indicate proteins linked to infertility. Bayesian FDR < 0.01 interactions by SAINT probability were considered high-confidence interactions.

In order to interrogate proteins interacting with WT and A189V mutant FSHR both in resting and ligand bound state, we utilized affinity purification combined with mass spectrometry (AP-MS). Due to the inherent nature of hPSCs to resist genetical manipulation, we used the Flp-In™ T-REx™ 293 cell line which allows rapid generation of isogenic and inducible stable cell clones with only a single copy of a transgene in their genome, which mimic the endogenous expression in our hPCS lines. HEK293 cells ectopically expressing WT or A189V FSHR were stimulated for 2 h with 100 ng/ml FSH prior to collection to study proteins interacting specifically with active receptors, whereas receptors at resting state were analyzed without preceding FSH stimulation. Altogether, 75 HCIs [(Bayesian FDR < 0.01) by SAINT probability] were identified, 27 of which were common to all four groups of bait (WT non-stimulated, WT FSH stimulated, A189V non-stimulated, and A189V FSH stimulated) as shown in Fig. 2 and Supplementary Table SII. There were 19 HCIs specific for WT receptors identified, of which 10 were specific for FSH stimulated and three for non-stimulated receptors. For the A189V FSHR 14 unique HCIs were identified, eight of which specific for the FSH stimulated receptor and one for the non-stimulated receptors (Fig. 2, Supplementary Table SII).

Proteins interacting with both WT and mutant FSHR irrespective of stimulation comprised several proteins involved in post-transcriptional processing and trafficking of proteins (Fig. 2). Many of the proteins could also be found among genes or proteins associated with male or female infertility in the curated Comparative Toxicogenomics Database (CTD) Gene-Disease Associations (Davis *et al.*, 2009) datasets using the Harmonizome (Rouillard *et al.*, 2016) (Fig. 2). Only WT FSHR was capable of binding FSH (CGA and FSHB), indicating that the same phenotype manifests in the HEK293 cells used in this method as in our iPSC-derived, differentiated cells.

Many of the HCIs specific for WT FSHR regardless of stimulation were mitochondrial proteins (Fig. 2). In addition to mitochondrial proteins, the RNA-binding protein YTH domain-containing family protein 3 (YTHDF3) interacted with non-stimulated WT FSHR, whereas e.g. a disintegrin and metalloproteinase with thrombospondin motifs 1 (ADAMTS1) interacted with FSH stimulated WT FSHR. Proteins interacting with A189V FSHR included several proteins involved in regulation of autophagy and apoptotic processes (Fig. 2). Protein sel-1 homolog 1 (SEL1L), a retrograde transport protein involved in retrotranslocation or dislocation of misfolded proteins from the ER for degradation (Sun *et al.*, 2014) was found to interact specifically with A189V FSHR upon FSH stimulation. Two of the proteins stably interacting specifically with A189V FSHR, amyloid precursor protein (APP) and TGF-beta receptor type-1 (TGFBR1), have been indicated to have a role in gonadal development or linked to infertility (Chen *et al.*, 2007; Silva *et al.*, 2015). Insulin-like growth factor 1 receptor (IGF1R), stromal cell-derived factor 2 (SDF2) and procadherin gamma-B5 (PCDHGB5) interacted specifically with both WT and A189V FSHR upon FSH stimulation (Fig. 2). Of these, IGF1R has previously been linked to fertility and plays a crucial role in FSH signaling in both male and female somatic gonadal cells (Baumgarten *et al.*, 2014; Law and Hunzicker-Dunn, 2016; Cannarella *et al.*, 2019; Neirijnck *et al.*, 2019).

## Discussion

In the present study, we have derived cells expressing endogenous *FSHR* that can be utilized to study the regulation and function of WT or mutant receptors in the physiological context of cell function. Expression of FSHR could be detected as early as Day 2 of differentiation, presumably at a time point roughly corresponding to the stage of primitive streak formation/gastrulation upon *invivo* development. Hence, FSH/FSHR may have a role already very early in human development. Indeed, FSHR expression has been detected in oocytes and preimplantation mouse and human embryos (Patsoula *et al.*, 2001; Meduri *et al.*, 2002; Patsoula *et al.*, 2003). FSH acts as an important survival factor on ovarian follicles (Yang and Rajamahendran, 2000; Zhou *et al.*, 2013) and, potentially, signaling through the FSHR may support the survival and/or proliferation of other cell types, as suggested for several epithelial cancer cells (Radu *et al.*, 2010; Lizneva *et al.*, 2019). The differentiation protocol used here aimed at inducing and promoting FSHR expression rather than generating a specific cell type. However, single-cell sequencing followed by cluster-based automatic cell annotation to characterize the cells based on their gene expression identified several of the cluster as gonadal cell types. Many were also identified as progenitor, fetal and stem cell types which may reflect the short differentiation period used. Although the protocol still needs to be optimized to increase the number of FSHR expressing cells, this can already be used for functional tests of the FSHR.

Cells expressing WT FSHR responded dose-dependently to FSH stimulation by increasing cAMP production and downregulating *FSHR* mRNA levels as previously shown in *invitro* conditions (Themmen *et al.*, 1991; Simoni *et al.*, 1997). Also, the expression of *INHA* and *StAR*, both previously shown to be induced by FSH (Feng *et al.*, 1989; Klaij *et al.*, 1990; Johnson and Bridgham, 2001), were upregulated. This indicates that FSHR expressed by the differentiated cells is biologically functional by activating the canonical, cAMP-mediated signaling pathway. In our *invitro* approach FSH furthermore directly upregulated expression of the non-canonical Notch ligand *DLK1* establishing a link between FSH/FSHR and DLK1 that, to our knowledge, has not been reported previously. In line with this, recent studies in primates have revealed that the NOTCH signaling ligand DLK1 is upregulated in juvenile Rhesus monkey testes after gonadotropin administration inducing puberty (Ramaswamy *et al.*, 2017). Interestingly, in adults *DLK1* is expressed in pituitary and ovaries (Canton *et al.*, 2019), and an inactivating *DLK1* mutation has been shown to be involved in central precocious puberty, suggesting its multiple roles in regulating reproductive functions (Dauber *et al.*, 2017; Gomes *et al.*, 2019).

As expected, the patient-derived cells expressing the mutant A189V *FSHR* were not able to respond to FSH stimulation by activating the canonical signaling pathway, as shown earlier in immortalized mouse Sertoli cells (Aittomaki *et al.*, 1995). This indicates that the differentiation protocol established here results in cells that can be utilized as a model for studying WT and mutant FSH receptors. Here, the inactivating A189V mutation was studied, but as the differentiation protocol can be applied to any hPSC line, other FSHR mutations and potentially also polymorphisms could be studied using either patient-derived iPSC lines or by utilizing, for example, CRIPSR/Cas9 technology for generating specific mutations in WT hPSC lines. FSHR polymorphisms have been associated with suboptimal ovarian stimulation in IVF treatments causing either unexpected hyperstimulation or poor response. However, because of their elusive underlying mechanisms (Alviggi *et al.*, 2018), the current differentiation protocol provides a useful tool for their further elucidation.

GPCRs have complicated and pleomorphic structures and functions and are therefore challenging to study. Recently, new interactions, locations, signaling mechanisms and functions have been found for several GPCRs, including the FSHR (Sayers and Hanyaloglu, 2018; Ulloa-Aguirre *et al.*, 2018).

PPI approaches have become increasingly utilized to identify new interacting partners for proteins of interest. Here, to reveal proteins stably interacting with FSHR, we overexpressed WT and A189V FSHR in HEK293 cells and applied AP-MS to detect proteins interacting with the WT and A189V receptor with or without FSH stimulation. HEK293 cells were used instead of differentiated hPSCs as the MAC-tag system requires extensive genetic manipulation difficult to establish in hPSCs, however, a system allowing expression levels mimicking the endogenous levels in our differentiated hPSC was used. Only one of the HCIs identified, Dolichyl-diphosphooligosaccharide–protein glycosyltransferase subunit DAD1 (DAD1), has to our knowledge previously been shown to directly interact with FSHR and several of them have been shown to be associated with gonadal function. However, previous interactions have mainly been detected in overexpression studies using viral promoters, whereas in the MAC-tag system only a single copy of the transgene is inserted in the genome, which mimic the endogenous expression. As expected, only WT FSHR was able to interact with FSHB and CGA. This further indicates that the mutant FSHR expression at the cell membrane was at any given time too low to allow detection of ligand binding. This also strengthens the findings that WT but not the A189V FSHR is translocated to the cell membrane (Aittomaki *et al.*, 1995; Rannikko *et al.*, 2002), although we were not able to confirm this due to lack of reliable antibodies.

Surprisingly, many of the proteins interacting with WT FSHR were mitochondrial proteins which may reflect FSHR-mediated processes such as steroid or lipid biosynthesis taking place in mitochondria. Although FSHR has not previously been thought to interact with mitochondrial proteins, recent studies have shown that some GPCRs can signal also in intracellular sites including mitochondria (Suofu *et al.*, 2017; Sayers and Hanyaloglu, 2018; Landomiel *et al.*, 2019). Whether these mitochondrial proteins play a functional role in FSHR signaling remains to be elucidated. Some endoplasmic reticulum (ER)-, Golgi- and lysosome-specific proteins were found to interact specifically with FSH stimulated WT FSHR. As these proteins interact specifically with WT and not with mutant FSHR, it may indicate that these proteins are involved in the possible failure of trafficking of the mutant FSHR to the plasma membrane. Interestingly, it has been shown that some newly synthesized GPCRs can be retained in the ER, and only upon stimulation are transported to the membrane (Dong *et al.*, 2007).

A novel ligand-induced interaction was discovered between WT FSHR and the metalloproteinase ADAMTS1. ADAMTS1 has been shown to play a role in follicular development and ovulation, and FSH stimulation of follicles leads to an increase of ADAMTS1 protein release (Shindo *et al.*, 2000; Richards *et al.*, 2005; Shozu *et al.*, 2005). Recently, insufficient ADAMTS1 expression was also reported to result in male infertility (Aydos *et al.*, 2019). Moreover, YTH domain-containing family protein 3 (YTHDF3) that recognizes and binds N6-methyladenosine (m6A)-containing RNAs (Shi *et al.*, 2017) specifically interacted with WT FSHR. Interestingly, in mice, m6A has been shown to be required for spermatogenesis and meiosis, and in zebrafish, the lack of m6A leads to reduced fertility and reduced FSHR levels in the testes (Xu *et al.*, 2017; Xia *et al.*, 2018). The direct interaction identified here between YTHDF3 and WT but not A189V FSHR indicates that methylation of specific residues may have an important role also in regulating human fertility, and that this process directly involves FSHR.

Several proteins interacting with the A189V FSHR upon FSH stimulation were also detected, strengthening the hypothesis that A189V FSHR can respond to stimulation although not binding the ligand at the plasma membrane, and might retain some G-protein independent signaling properties. Although testing multiple antibodies targeting FSHR, we were not able to confirm differential localization of WT and A189V FSHR with, for example, immunocytochemistry due to non-specificity of the tested antibodies. We cannot exclude the possibility that a minute fraction of the A189V FSHR is transiently expressed on the plasma membrane, possibly activating other pathways responding more rapidly to stimulus than the canonical cAMP-dependent pathway before being reinternalized. This is however unlikely, as our sensitive PPI analysis showed no interaction between A189V FSHR and FSH.

IGF1R, which in our study interacted with both WT and A189V FSHR exclusively upon FSH stimulation, has been shown to be required for FSH signaling in mouse and human granulosa cells (Zhou *et al.*, 2013; Law and Hunzicker-Dunn, 2016), and in porcine neonatal and rat immature Sertoli cells (Khan *et al.*, 2002; Cannarella *et al.*, 2018). Insulin/IGF signaling via IGF1R has been shown to be required for both male and female fertility and development of gonadal somatic cells, and to be necessary for the FSH induced proliferation of immature Sertoli cells and proliferation and differentiation of granulosa cells (Pitetti *et al.*, 2013; Baumgarten *et al.*, 2014, 2017). The mechanism by which A189V FSHR can interact with IGF1R and possibly affect signaling remains to be elucidated. Intriguingly, IGF1R has been shown to act as a transcriptional activator upon translocation to the nucleus, in addition to its role as a cell surface receptor (Sarfstein and Werner, 2013), which may explain the interaction with A189V FSHR. Some of the proteins specifically interacting only with the A189V FSHR upon stimulation are linked to regulation of apoptosis or autophagy, which may reflect disturbed receptor trafficking and accumulation of misfolded proteins.

In conclusion, we have generated cells endogenously expressing either WT or A189V mutant FSHR. This enables signaling pathway studies in cells carrying mutations or polymorphisms possibly affecting the function of FSHR. Utilizing protein interactomics approach, we have shown that the WT and the A189V FSHR interact with distinct proteins and that FSH stimulation affects the interactions of both. Further studies are required to understand how FSH triggers the diverse downstream signaling when predominantly residing inside the cell.

## Supplementary data

Supplementary data are available at *Molecular Human Reproduction* online.

## Supplementary Material

gaac012_Supplementary_Data

## Data Availability

The data underlying this article will be shared on reasonable request to the corresponding author. Single-cell RNA sequencing data and expression matrix of filtered and normalized single-cell RNA sequencing data has been deposited in NCBI’s Gene Expression Omnibus (GEO) and can be accessed through GEO Series accession number GSE184352 (https://www.ncbi.nlm.nih.gov/geo/query/acc.cgi?acc=GSE184352). Mass spectrometry data has been deposited to MassIVE (https://massive.ucsd.edu/) with the identifier: MSV000088577.
