# Supplementary material for: Human pluripotent stem cell-derived cells endogenously expressing follicle-stimulating hormone receptors: modeling the function of an inactivating receptor mutation
Source: Mol Hum Reprod. 2022 Apr 26;28(5):gaac012. doi: 10.1093/molehr/gaac012 (PMC9308958; doi:10.1093/molehr/gaac012)
Supplement: gaac012_Supplementary_Data [file gaac012_supplementary_data.zip › SI.pdf]

Supplementary Information

Human pluripotent stem cell-derived cells endogenously expressing follicle-stimulating hormone receptors: modeling the function of an inactivating receptor mutation

Karolina Lundin, Kirsi Sepponen, Pia Väyrynen, Xiaonan Liu, Dawit A. Yohannes, Mantas Survila, Bishwa Ghimire, Johanna Känsäkoski, Shintaro Katayama, Juha Partanen, Sanna Vuoristo, Pauliina Paloviita, Nafis Rahman, Taneli Raivio, Kaisu Luiro, Ilpo Huhtaniemi, Markku Varjosalo, Timo Tuuri, and Juha S. Tapanainen.

Correspondence: juha.tapanainen@helsinki.fi

List of supplementary figures:

Supplementary Figure 1: Characterization of differentiated H9 (WT) hES cells and HEL127.6 and HEL128.5 (A189V FSHR mutant) patient-derived hiPS cells.

List of supplementary tables submitted as separate Microsoft Excel files:

Supplementary Table I: Single Cell RNAseq Read and Quality Control Statistics

Supplementary Table II: List of all detected high confidence interactions (Bayesian FDR ≤ 0.05) by SAINT probability

Links to data submitted to repositories:

Single cell RNAsequencing raw data as well as a txt file containing expression matrix of filtered and normalized (scaled and log transformed) data has been deposited in NCBI's Gene Expression Omnibus (GEO) and is accessible through GEO Series accession number GSE184352 (https://www.ncbi.nlm.nih.gov/geo/query/acc.cgi?acc=GSE184352).

Mass spectrometry data were deposited to MassIVE (https://massive.ucsd.edu/) with the identifier: MSV000088577.

Fig S1.

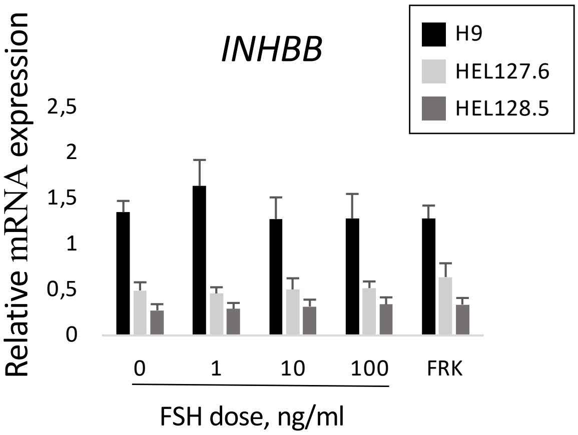

Supplementary Figure 1. Characterization of differentiated H9 (WT) hES cells and HEL127.6 and HEL128.5 (A189V FSHR mutant) patient-derived hiPS cells. 8 hours of stimulation with increasing doses of FSH or 10 μM forskolin at day 8 of differentiation did not affect the expression levels of *Inhibin Subunit Beta B* (*INHBB*) in neither WT cells (black bars) nor in A189V mutant lines (light and dark grey bars), when compared to undifferentiated cells. The values represent mean +/- standard error of mean from three independent experiments. No significant differences were detected (one-way ANOVA).
